# Supplementary material for: Association between oral microbiome and seven types of cancers in East Asian population: a two-sample Mendelian randomization analysis
Source: Front Mol Biosci. 2023 Nov 21;10:1327893. doi: 10.3389/fmolb.2023.1327893 (PMC10702768; doi:10.3389/fmolb.2023.1327893)
Supplement: Supplementary file 1 [file DataSheet1.ZIP › Supplementary Materials/MR plots of saliva/saliva═╝/Prostate cancer/pheno.2198_to_prostate cancer_forest.pdf]

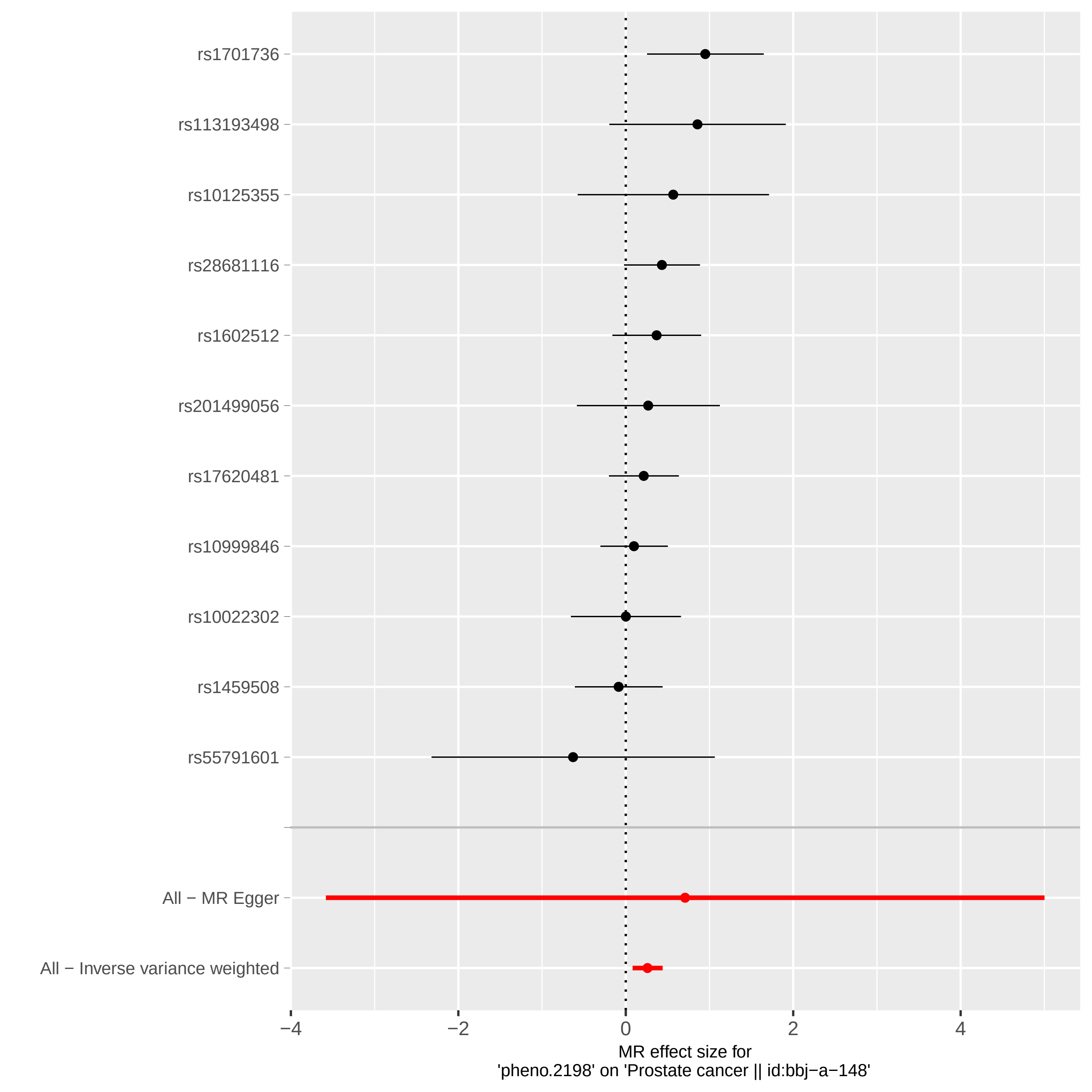
rs1701736
rs113193498
rs10125355
rs28681116
rs1602512
rs201499056
rs17620481
rs10999846
rs10022302
rs1459508
rs55791601
All − MR Egger
All − Inverse variance weighted
−4
−2
0
2
4
MR effect size for
'pheno.2198' on 'Prostate cancer || id:bbj−a−148'
